# Defining the Newborn Blood Spot Screening Reference Interval for TSH: Impact of Ethnicity

**DOI:** 10.1210/jc.2016-1822

**Published:** 2016-07-11

**Authors:** Catherine Peters, Ivan Brooke, Simon Heales, Adeboye Ifederu, Shirley Langham, Peter Hindmarsh, Tim J. Cole

**Affiliations:** Department of Endocrinology (C.P., S.L., P.H.), and Chemical Pathology, Laboratory Medicine (I.B., S.H., A.I.), Great Ormond Street Hospital, London WC1N 3JH, United Kingdom; and Population, Policy, and Practice Programme (T.J.C.), UCL Institute of Health, London WC1N 1EH, United Kingdom

## Abstract

**Context:**

There is variability in the congenital hypothyroidism (CH) newborn screening TSH cutoff across the United Kingdom.

**Objective:**

To determine the influences of year, gender, and ethnicity on screening variability and examine whether there is an optimal operational TSH cutoff.

**Design and Setting:**

Single center, retrospective population study using blood spot TSH cards received by the Great Ormond Street Hospital Screening Laboratory between 2006 and 2012.

**Patients:**

A total of 824 588 newborn screening blood spot TSH cards.

**Intervention:**

Blood spot TSH results were recorded with demographic data including the Ethnic Category Code.

**Main Outcome Measures:**

The proportions of samples exceeding different TSH cutoffs, ranked by ethnicity.

**Results:**

The proportion of samples exceeding the TSH cutoff increased over time, with the cutoff at 4 mU/L, but not at 6 mU/L. There was a consistent trend with ethnicity, irrespective of cutoff, with the odds ratio of exceeding the TSH cutoff lowest (∼1.0) in White babies, higher in Pakistani and Bangladeshi (>2.0), and highest in Chinese (>3.5).

**Conclusions:**

The blood spot TSH screening data demonstrate a clear ranking according to ethnicity for differences in mean TSH. This suggests that there may be ethnic differences in thyroid physiology. Ethnic diversity within populations needs to be considered when establishing and interpreting screening TSH cutoffs.

## Abstract (precis)

Newborn blood spot TSH screening for congenital hypothyroidism within a population of London, UK demonstrates ethnic ranking for differences in mean TSH with higher TSH results in Asian infants.

The UK newborn blood spot screening program for congenital hypothyroidism (CH) was established in 1983 (1). It has led to the diagnosis and early levothyroxine treatment for thousands of babies with CH who might otherwise have developed profound neurodevelopmental impairment.

The UK screening program for CH is based on the measurement of TSH concentration in a heel-prick blood spot taken at 5–10 days of age. Babies whose TSH concentration exceeds 20 mU/L are considered to be screen positive and require immediate referral for venous thyroid function tests and clinical evaluation. A lower borderline TSH cutoff was introduced to minimize false-positive notifications due to a delayed physiological TSH surge. It was assumed that such babies would normalize TSH over the following week. Therefore, in babies with an initial blood spot screen between the lower cutoff and 19.9 mU/L, a second sample is taken 7 days after the first. Cases that remain abnormal are referred for venous testing and clinical evaluation (1).

The value of this lower TSH cutoff has been the subject of much debate because differences have arisen over time between screening laboratories across the United Kingdom and internationally (2–4). This has occurred in response to differences and changes in TSH assays, reports of missed cases of CH, and the rigor with which the normal reference values were constructed (5). The lower cutoff in our center is currently 6 mU/L.

There is a lack of evidence to assess the optimal lower cutoff point in terms of screening sensitivity and specificity. In addition, UK children vary between regions in terms of ethnicity and iodine sufficiency, which may also influence the optimal cutoff value.

To address these issues, we examined screening data for the whole newborn population from a single laboratory in North London over a 7-year period. We aimed to document changes in TSH concentration over time and the differences between ethnic groups.

## Subjects and Methods

Between January 2006 and December 2012, 824 588 babies from the North Thames region (which includes Greater London and parts of the counties of Bedfordshire, Essex, and Hertfordshire) were screened for CH at the Great Ormond Street Hospital (GOSH) Newborn Blood Spot Screening Laboratory.

The biochemical results were recorded along with demographic data, including gestational age. Ethnicity was coded using the Ethnic Category Code published by the Health & Social Care Information Centre (6). Percentages by ethnic group were compared with the London and overall UK populations using data from the 2011 UK census (7).

### Assays

Blood spot TSH concentrations were measured by the automated dissociation enhanced lanthanide fluoroimmunoassay (AutoDELFIA) (Perkin Elmer) system. The method is calibrated using six standards with values ranging from < 1 mU/L to 300 mU/L, with some variation for each kit lot. The interbatch precision of this method ranges from 6 to 8%, with no trend over the range of values. TSH in dried blood spot specimens has been shown to be stable for at least 1 month at room temperature. If stored at 4°C with desiccant, there is no degradation of TSH for at least 1 year. All samples in this study were analyzed within 1 month of sample collection. There was no modification or change to the assay procedure during the study period, and no assay drift was reported. The assay was enrolled in the UK External Quality Assurance Scheme, and reports were satisfactory throughout this period.

### Statistics

The blood spot TSH data were tabulated by ethnic group, year, sex, and integer TSH value. Binomial models were then fitted to the counts using weighted logistic regression, to estimate the effects of ethnicity, year, and sex on the chance of TSH exceeding a prespecified cutoff point, for integer cutoffs between 4 and 10 mU/L.

The ethnic group-specific odds ratios of the TSH exceeding each cutoff, adjusted for year and sex, were then plotted to show how the ranking of ethnic groups varied by cutoff point, with White British babies as the reference.

The χ^2^ test was used to compare the frequency of ethnic groups in the various population groups. Trends for the number screened per annum were assessed using Pearson's correlation coefficient.

## Results

A total of 824 588 newborn babies from North Thames were screened for CH at GOSH between January 2006 and December 2012 (Table 1). The numbers rose significantly over time (r = 0.93; *P* = .002) with an extra 1700 babies per year. Overall, there were 47% girls, 49% boys, and 4% sex not recorded. The UK screening program assumes potential immaturity of the thyroid axis in infants < 32 weeks old, and a second card is requested 4 weeks later in those with a normal TSH result. Fewer than 1.4% of the cohort were recorded to have a gestational age ≤ 32 + 6 weeks, and the ethnic distribution within this group was similar to the cohort as a whole.

**Table 1.** Numbers of First Newborn Blood Spot Screening Cards by Year

Year	2006	2007	2008	2009	2010	2011	2012
Card no.	110174	115882	117341	118758	119870	120449	122114

**Figure 1.**
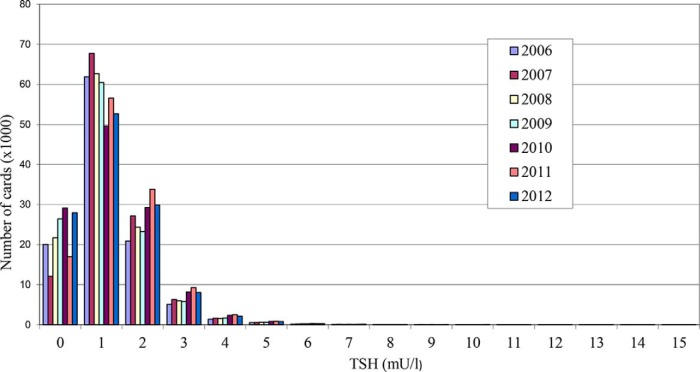
Distribution of blood spot TSH concentrations for the years 2006–2012.

The percentages of babies with blood spot TSH concentrations exceeding specific cutoffs are shown in Table 2. Adjusted for ethnicity and sex, the proportion of samples exceeding a cutoff point of 4 mU/L on the first card rose significantly over time (*P* < .0001), but with cutoffs of 6, 8, or 10 mU/L there was no such trend (*P* > .1). Thus, the proportion of samples between 4 and 6 mU/L increased over time, but over 6 mU/L it was stable (Figure 1). Conversely, adjusted for ethnicity and year, there was no difference between males and females in the proportion exceeding cutoffs 4, 6, or 8 mU/L, but there was a small female excess for 10 mU/L (*P* = .0003).

**Table 2.** Overall Percentages of TSH Exceeding Various Cutoff Points

Cutoff Point	4 mU/L	5 mU/L	6 mU/L	8 mU/L	10 mU/L
% Exceeding cutoff point	1.06	0.49	0.30	0.16	0.10

Table 3 shows the percentages by ethnic group in the GOSH screening samples and subsequent referrals in 2011 compared to the 2011 UK census (7). The census showed a significantly different ethnic distribution in London compared to the United Kingdom (*P* = .01). The ethnic distribution of blood spot samples received by the GOSH Laboratory was similar to London 2011, apart from a slight excess of “Other”. Compared to the laboratory-screened group, there was an excess of Asian/Asian British patients referred to GOSH for further evaluation and treatment (n = 138) (*P* = .01).

**Table 3.** Percentages by Ethnic Group in the Great Ormond Street Hospital Screening and Referral for Treatment Populations Compared to UK and London 2011 Census Figures

Ethnic Group	UK 2011 Census	London 2011 Census	GOSH Laboratory 2011	GOSH Referrals 2011 (n = 138)
White	86	60	53	44
Asian/Asian British	7.5	18	19	34
Black/Black British/African/Caribbean	3.3	13	10	9.2
Mixed	2.2	5.0	6.0	2.8
Other	1.0	3.4	12	7.8

Data are expressed as percentages.

**Figure 2.**
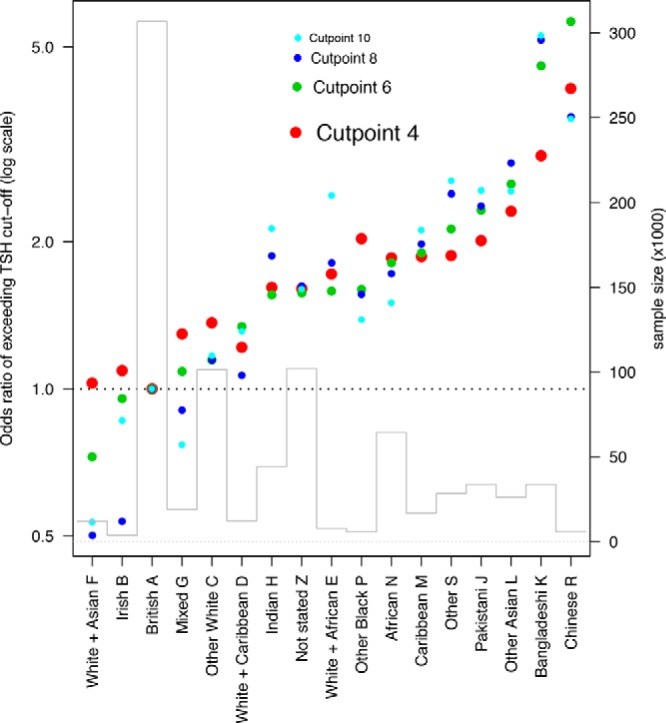
Odds ratio of exceeding a blood spot screening cutoff with the British White population set at 1.0. The sample size in each ethnic group is shown by the block histogram. Ethnic code categories are represented by letters according to Office for National Statistics classification (7).

Figure 2 indicates the ethnic breakdown in 17 categories including “not stated.” The histogram shows the group sample sizes, covering an 80-fold range from White British (n = 306 708) to White Irish (n = 3760). The ethnic groups were ranked by the proportion exceeding a TSH cutoff point of 6 mU/L (the screening cutoff used by GOSH), adjusted for year and sex, and Figure 2 also shows the odds ratio of exceeding the 6 mU/L cutoff in each ethnic group compared to White British babies, ranked from low to high (green points). Similar results, distinguished by color and point size, are shown for cutoff points of 4, 8, and 10 mU/L. As the cutoff point rises, the screen-in prevalence falls, reflected by the points for the higher cutoffs becoming progressively smaller in diameter. The graph shows a consistent trend, irrespective of cutoff value, with a low group (predominantly White) near 1, a middle group (Indian, African, and Caribbean) above 1.5, and a high group (Pakistani, Bangladeshi, and Chinese) exceeding 2. Note that all of the Chinese odds ratios exceed 3.5.

## Discussion

Newborn screening TSH cutoff points differ between centers nationally and internationally. There is no clear consensus on the rationale for the cutoff points, which are based on historical opinion, differences in biochemical assays, and missed cases of CH. The UK 2011 population census data showed differences in the ethnic distribution in London compared to the United Kingdom as a whole. The GOSH screening laboratory ethnicity data reflect this London distribution, and the diversity has provided an opportunity to examine the effect of ethnicity on the TSH cutoff point.

We examined TSH cutoffs at integer values from 4 to 10 mU/L. For cutoffs of 6 and 8 mU/L, there was no trend over time and no gender difference, so in this sense these cutoffs are optimal. Figure 2 demonstrates that the odds ratios of TSH exceeding the cutoff vary highly significantly by ethnicity. For the lowest cutoff point (4 mU/L, red points), the odds ratios are relatively close to 1 (the White British reference), but as the cutoff point rises, they move progressively away from it. This suggests that the ethnic differences are due to a shift in mean TSH, so the further one goes into the tail of the distribution, the more pronounced the ethnic differences are. These results imply that there are ethnic differences in thyroid physiology, which may be due to genetic factors and which should be considered when examining TSH cutoffs in different populations. Consanguinity is not recorded on the newborn screening cards, but this would be relevant if there were genetic differences.

The ethnic groups with the greatest shifts in mean TSH concentration originate from regions of the world with historical records of iodine deficiency (8). There is increasing evidence that the UK population of young women is iodine insufficient (9–11), which may compound this effect. Previous attempts to link newborn screening TSH data to iodine have had mixed results. The World Health Organization (WHO) previously suggested that a population frequency of < 3% with a TSH > 5 mU/L at 3–4 days of age was indicative of iodine sufficiency in a population (12). The introduction of iodinated salt in Poland reduced the prevalence of neonatal TSH > 5 mU/L from 20 to 5% over 5 years (13). However, the WHO definition is controversial, with limitations based on the day of sampling and the assay sensitivity (14).

There is a need to examine iodine sufficiency of UK newborns in more detail. Our data showed only 1% exceeding a TSH cutoff point of 4 mU/L, although screening occurs later at 5–10 days. Babies with raised TSH concentrations secondary to iodine insufficiency have a preventable cause of CH. This has neurodevelopmental implications (10) and, although the hypothyroidism may be transient if the baby is subsequently given iodine-fortified milk formula, there remains a group who may have chronic iodine insufficiency, mild CH, and an ongoing need for T_4_ therapy.

It is unlikely that ethnic differences can be explained by differences in the postnatal TSH surge. In this dataset, screening takes place at 5–10 days of age. Babies with a borderline result of 6–19.9 mU/L have a second screening card taken 7 days after the first and are referred if it is > 6 mU/L. It is assumed that this second card would have normalized in those babies with a delayed TSH surge. We did not look at second card data, but the increase in clinical referrals for Asian babies implies that this ethnic effect persists and reflects an alternative physiological effect (2).

This newborn screening dataset demonstrates a yearly increase in babies screened, reflecting the rising birth rate in this geographical area. The study has various strengths. The population used is large and unselected, and hence is representative of normal newborns. The effects of illness and prematurity are small in this population. The age of the population is well defined between 5 and 10 days of postnatal life, and specimen collection and processing was standardized. Analysis was undertaken in a single laboratory using the same assay methodology. Assay performance did not change significantly over time. Finally, the sample size was sufficient to detect differences between ethnic groups. The population mix in the London area differs from other regions of the United Kingdom (Table 3), and our data demonstrate that the ethnicity of babies born within the GOSH screening catchment is representative of the London population.

As stated above, the best cutoff to use would be either 6 or 8 mU/L, although this conclusion is based on year and gender differences rather than screening sensitivity and specificity. Screening sensitivity and specificity for CH are based on long-term neurodevelopmental outcomes in the untreated/late-treated child, and data exist only for historical cases of severe CH. However, this large dataset from a single center based in an ethnically diverse city provides new insights into the effects of ethnicity on screening TSH concentrations, and this needs to be considered when setting cutoff points. It also suggests that subtle variation in thyroid physiology and/or iodine sufficiency may account for differences in the reported incidence of CH and in particular for the increase in “milder” forms of dyshormonogenesis.
